# The relationship between mandatory exercise and exercise habits in Chinese colleges and universities: an analysis of multiple mediating effects

**DOI:** 10.3389/fpsyg.2025.1536487

**Published:** 2025-04-25

**Authors:** Xiangxuan Guo, Dawei Cao, Dong Wu, Lulu Kuang

**Affiliations:** ^1^Chinese Wushu Academy, Beijing Sport University, Beijing, China; ^2^School of Physical Education, Huaibei Normal University, Huaibei, China; ^3^School of Physical Education, Shanxi Normal University, Xi'an, China

**Keywords:** college and university, mandatory exercise, exercise habits, self-efficacy, exercise attitudes

## Abstract

This study aims to explore the relationship between mandatory exercise and exercise habits in colleges and universities and to investigate the multiple mediating effects of self-efficacy and exercise attitudes. The study adopted convenience sampling, with 589 junior students as the research subjects, and conducted a questionnaire survey using professional scales. The results show that mandatory exercise in colleges and universities has a significant positive effect on the formation of exercise habits. Self-efficacy and exercise attitudes play a significant mediating role and chain mediating effect between mandatory exercise and exercise habits, with the direct effect being the most significant, followed by the chaining effect, the mediating effect of self-efficacy, and the mediating effect of exercise attitudes. This study further reveals the internal mechanism underlying the relationship between mandatory exercise and exercise habits, which is highly important for promoting the development of “exercise habits,” i.e., the overall development of college and university students.

## Introduction

With the development of society and changes in lifestyle, the physical fitness of college and university students has increasingly become a concern. Studies have shown that exercise has a significant positive effect on improving students’ physical fitness. However, facing the explosive era of the internet and entertainment, college and university students generally lack interest and habits in physical exercise, leading to a continuous decline in physical fitness. Therefore, how colleges and universities can effectively promote student exercise and develop good exercise habits has become an urgent problem to be solved. To promote the development of college and university students’ exercise habits, some colleges and universities have adopted the method of “mandatory exercise,” that is, through administrative mandatory measures and the supervision of teachers to ensure student participation in physical activities. For example, Southwest-Associated Universities implemented 1 hour of mandatory exercise per day, locking libraries, dormitories, and classrooms to compel students to participate in physical activities. From 1911 to 1918, Tsinghua University’s “mandatory exercise” had a positive effect on the cultivation of high-quality talents with all-round personalities, the development of modern sports, and the core of Tsinghua’s sports culture (Hu X. S., 2021). Modern colleges and universities have implemented policies such as “Sunshine Run,” “Le Run,” and “Morning Run.” These mandatory measures have achieved good results in history and reality, helping to improve students’ physical fitness, optimize their qualities, and enhance their self-efficacy. They can be used as a means of physical and mental promotion intervention, helping students accumulate and expand their perceptions of sports, correct negative sports concepts, be conscious, and exercise attitudes. Additionally, research on “mandatory exercise” in China has focused mainly on historical and sociological dimensions (Hu Y., 2021; Zhang, 2014; Gu, 1994), clinical medicine and life sciences (Zhong et al., 2009; Wang and Huang, 2006; Liu and Liu, 2007; Xu et al., 2006; Pang et al., 2010; Mo and Shi, 2020; Li et al., 2008), and psychology (Wang et al., 2021; Wang et al., 2022; Yang, 2014), with psychological research primarily concentrating on self-esteem and interest. Internationally, the focus is on clinical medicine (Meyer et al., 2011; Taranis et al., 2011; Plateau et al., 2017), sociology (Hallward and Duncan, 2021; Goodwin et al., 2014; Reynolds et al., 2023), behavioral science (Lichtenstein et al., 2017; Solfrid et al., 2020), psychology (Palermo and Rancourt, 2023; Pike et al., 2022), sports and exercise science (Limburg et al., 2021), life sciences (Ghonsulakandi et al., 2017), and basic and applied research (Ahn et al., 2020; Fioravanti et al., 2023). Psychological research on exercise has focused mainly on anxiety (Cuesta-Zamora et al., 2023), body image (Palermo and Rancourt, 2023), exercise identity (Martin and Racine, 2017), emotional regulation (Goodwin et al., 2012), depression (Weinstein et al., 2015), social relationships (Patterso et al., 2022), self-compassion (Cuesta-Zamora et al., 2022), personality (Goodwin et al., 2011), sex (Wischenka and Swencionis, 2019), self-schema (Patterson and Goodson, 2018), meditation (Segrave et al., 2022), and mental health (Cosh et al., 2023). Research on the relationships between mandatory exercise and self-efficacy, exercise attitudes, and exercise habits is still in its infancy. However, based on common sense, it can be inferred that there is a dual mechanism by which external coercive systems affect the self-efficacy and attitude of exercisers. Institutional design needs to balance external norms and internal motivation, and promote exercise habits through a sense of efficacy or exercise attitude. Therefore, this study is based on the relationship between mandatory exercise and exercise habits and explores the multiple mediating effects of self-efficacy and exercise attitudes.

## Theoretical analysis frameworks

In psychology, there is a famous theory called “habit formation theory,” which suggests that habits are gradually formed through repeated behaviors (Verplanken and Wood, 2006). When an individual repeatedly performs a certain behavior, the neural pathways in the brain gradually strengthen, eventually forming habits. This process requires time and patience, as the ancient Greek philosopher Aristotle believed: “We are what we repeatedly do. Therefore, excellence is not an act, but a habit.”

Compulsion and habit formation are complex processes involving the repetition of individual behaviors and psychological adaptation. As the British philosopher John Locke said, “Habits, if not the best servants, are the worst masters.” This statement emphasizes the double-edged nature of habits, which can become a powerful force to promote human progress or may become a shackle that binds us. Grasping and effectively utilizing the relationship between compulsion and habits can promote human progress and development. Mandatory behavior is often an extreme manifestation of the process of habit formation. When an individual repeatedly performs a certain behavior unwillingly in a policy environment, this behavior may become mandatory. At this time, the individual may feel anxious or uncomfortable unless the behavior is performed. A psychology viewpoint suggests that mandatory behavior may be a coping mechanism to alleviate inner unease or pressure (Hu Y., 2021). In summary, habit formation is a gradual process that requires individuals to consciously repeat behaviors and maintain patience and perseverance during the process. Moreover, the emergence of mandatory behavior should be utilized to learn to cope with stress and challenges in a healthy way.

**Figure 1:**
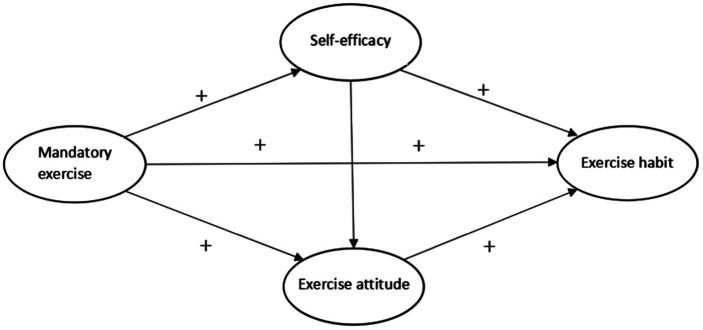
Framework for the analysis of the relationships between mandatory exercise and college and university students’ exercise habits.

Colleges and universities use their strong sports environment and cultural policy to encourage students to form exercise habits. This change caused by environmental pressure is a result of the joint action of initiative and passivity. Mandatory exercise focuses not only on the imparting of skills but also on the cultivation of students’ “complete personality.” The mastery of these sports skills, combined with the awareness and habits of physical exercise, constitutes an indispensable part of students’ lives later, having an important impact on their work and life. For example, Tsinghua University’s “mandatory exercise” policy and sports tradition effectively promoted the formation of students’ exercise habits through environmental influence, curriculum setting, the cultivation of sports spirit. This resulted in a rational sense of self-efficacy and a positive exercise attitude, thus had a profound impact on students’ physical and mental health and future development. The physical education teacher Mr. John MA emphasized that the effect of sports lies in cultivating personality, making up for the insufficiency of education, teaching students how to protect their bodies, and cultivating a positive spirit. Tsinghua University President Nanxiang JIANG’s slogan “Work for the motherland healthy for 55 years” reflects Tsinghua’s concept and attitude of combining sports with healthy work, affirming the positive significance of sports for individual self-efficacy. This concept encourages students to form the correct exercise attitude and develop good exercise habits to maintain a healthy body and vigorous energy. Therefore, the function of mandatory exercise is first reflected in physical exercise and repetition, which promote the formation of habits in people and the production of positive self-efficacy and exercise attitudes, which are important for people. Therefore, colleges and universities can promote the formation of “exercise habits” through the implementation of “mandatory exercise” to achieve this through the mediating effects of “self-efficacy” and “exercise attitudes.” Based on the above discussion, the following theoretical analysis framework is formed (Figure 1).

From the analysis framework shown in Figure 1. On the one hand, colleges and universities implement “mandatory exercise” policies to increase their self-efficacy, and at the same time, they guide students to develop a positive “exercise attitude” in physical exercise, thereby forming exercise habits on this basis. On the other hand, in the process of implementing “mandatory exercise” policies in colleges and universities, not only can self-efficacy be increased, but students’ identification with sports can also be promoted, deepening the purpose of “mandatory exercise” in promoting the development of exercise habits. Based on the above analysis, the following research hypotheses are proposed:

*H1*: Colleges’ and universities’ “mandatory exercise” policies positively predict the development of students’ exercise habits.

*H2*: Colleges’ and universities’ “mandatory exercise” policies indirectly promote the formation of students’ exercise habits through the separate mediating effects of “self-efficacy” and “exercise attitudes.”

*H3*: Colleges’ and universities’ “mandatory exercise” policies indirectly promote the formation of students’ exercise habits through the chain mediating effects of “self-efficacy” and “exercise attitudes.”

## Research data and methods

### Data used

To understand the multiple mediating effects of self-efficacy and exercise attitudes on the relationship between mandatory exercise and exercise habits, the research team developed the “The Relationship between mandatory exercise and Exercise Habits: Multiple Mediating Effects Analysis” questionnaire. The questionnaire includes the mandatory exercise Test (CET), self-efficacy items, exercise attitude scales, and exercise habit items. The questionnaire survey was conducted through convenience sampling, and electronic questionnaires were pushed online for college and university students to complete. The survey sample included 597 third-year students selected from H college and university (The survey group has completed 2 years of compulsory exercise in accordance with school regulations). This study removed cases with missing information and those that did not make sense, and the final valid sample was 589, with a valid recovery rate of 98.66%. Among the valid survey samples, males accounted for 47.03% and females accounted for 52.97%; Humanities majors account for 51.1%, while science majors account for 48.9%; Urban students account for 46.18%, while rural students account for 53.82%.

### Variable selection

#### Dependent variable

The dependent variable in this study was exercise habits. The examination of the subjects’ exercise habits included an examination of their exercise habits and the frequency of exercise. Drawing on the international criteria for the concept of the “sports population” (Qiu, 1999), the standard for having an exercise habit is as follows: exercise frequency ≥1 time/week, and each exercise lasting more than 20 min is considered “yes,” and vice versa, it is considered “no.” According to the relevant standards of the Healthy China Action Promotion Committee (2019), those who participate in physical exercise three times a week or more are considered too frequently participate in physical exercise, so the exercise frequency is divided into ≤3 times/week and >3 times/week. The evaluation standard is divided into three levels: never (=1), low frequency (=2), and high frequency (=3). The higher the score is, the greater the exercise frequency.

#### Independent variable

The independent variable in this study was mandatory exercise. Mandatory exercise is measured via the mandatory exercise Test (CET) developed by Taranis et al. (2011). The scale includes 24 items and consists of 5 subscales, namely, avoidance and rule-driven behavior, exercise for weight control, mood improvement, lack of exercise pleasure, and exercise rigidity. The scale uses a six-point Likert scale: never (=0), occasionally (=1), sometimes (=2), often (=3), frequently (=4), and always (=5). The mandatory exercise Scale has been widely used in clinical and scientific research, and the internal consistency coefficient (Cronbach’s alpha) of the English version of the scale is approximately 0.85 ~ 0.88 across different samples. After being translated into Chinese, the internal consistency coefficient of the scale was 0.82 (Wang et al., 2022).

#### Mediating variables

The mediating variables in this study are self-efficacy and exercise attitudes. Drawing on the research of scholars Li et al. (2012), McHeill et al. (2006), and Su (2004), a self-efficacy of 3 items is determined: “Confident to persist in participating in exercise,” “Confident to overcome unfavorable factors that hinder participation in exercise,” and “Willing to make efforts to continue to exercise in the future” (Liu et al., 2024). The study uses a 5-point Likert scale ranging from “completely disagree” to “completely agree,” with scores ranging from 1 ~ 5 points. The higher the score is, the greater the exercise self-efficacy. The exercise attitude measurement used Mao's (2003) “Exercise Attitude Scale.” The scale includes 8 dimensions, namely, behavioral attitude, goal attitude, behavioral cognition, behavioral habits, behavioral intention, emotional experience, sense of behavioral control, and subjective standards, with a total of 70 questions. The scale structure model x2/df = 3.67, NNFI = 0.93, CFI = 0.94, AGFI = 0.87, RMSEA = 0.06, and the Cronbach’s alpha for each dimension is 0.64 ~ 0.89 (Mao, 2003; Zhang and Mao, 2004). The answer options include completely disagree (=1), disagree (=2), not sure (=3), agree (=4), and completely agree (=5). This study sums the scores of each dimension to obtain the score of each dimension of exercise attitudes, and the higher the score is, the greater the degree of recognition of each dimension of exercise attitudes.

#### Control variables

The control variables involved in the model are mainly sex and hobbies. In the sex item, “1” represents male, and “2” represents female; in the hobby item, “1” represents sports enthusiasts, and “2” represents non-sports enthusiasts.

### Statistical methods

Descriptive statistical analysis methods were used to analyze the basic profile of the sample, the development of mandatory exercise, and its impact on college and university students’ exercise habits. This study also uses Hayes’s multiple mediating effect analysis method to test the mediating effects of self-efficacy and exercise attitudes. First, Hayes’s SPSS macro Model 6 (Hayes, 2013) is used to estimate the mediating effects of self-efficacy and exercise attitudes on the relationship between college and university mandatory exercise and students’ exercise habits. Moreover, the bootstrap method is used, which is based on 5,000 random samples to estimate the 95% confidence interval, to test the significance of the mediating effects. If the 95% confidence interval does not include 0, the difference is statistically significant. The fitted equation model is as follows.

Total effect:

$$Y=\lambda_{0}+\lambda_{1}X+\lambda_{2}C_{1}\text{,}$$

Multiple mediating effects:

$$M_{a}=\beta_{0}+\beta_{1}X+\beta_{2}M+\beta_{3}C_{2}\text{,}$$

$$M_{b}={\beta^{’}}_{0}+{\beta^{’}}_{1}X+{\beta^{’}}_{2}\;W+{\beta^{’}}_{3}C_{2}\text{,}$$

$$M_{c}={\beta^{”}}_{0}+{\beta^{”}}_{1}X+{\beta^{”}}_{2}\;M+{\beta^{”}}_{3}\;W+{\beta^{”}}_{4}C_{2}$$

In the above formulas, X represents the independent variable, Y represents the dependent variable, M and W represent the mediating variables, and C_1_ and C_2_ represent control variables. λ_0_ and β_0_ represent the intercepts; λ_1_, β_1_, β’_1,_ and β”_1_ represent the impacts of the independent variable X on the dependent variable Y; and λ_2_, β_3_, β’_3,_ and β”_4_ represent the impacts of the control variables on the dependent variable. In addition, the β_2_, β’_2_, β”_2_, and β”_3_ parameter tests represent mediating effects, indicating the extent to which the impact of the independent variable on the dependent variable is affected by the mediating variables. Multiple mediating effects should include both independent mediating effects and chain mediating effects (Kong and Wang, 2022). The independent mediating effects were “mandatory exercise–self-efficacy–exercise habits” and “mandatory exercise–exercise attitude–exercise habits.” The chain mediating effects are “mandatory exercise–self-efficacy–exercise attitudes–exercise habits,” and the total mediating effect is obtained by adding the two mediating effects.

## Mandatory exercise policies of colleges and universities and their implementation

### Mandatory exercise policies of colleges and universities

**Figure 2:**
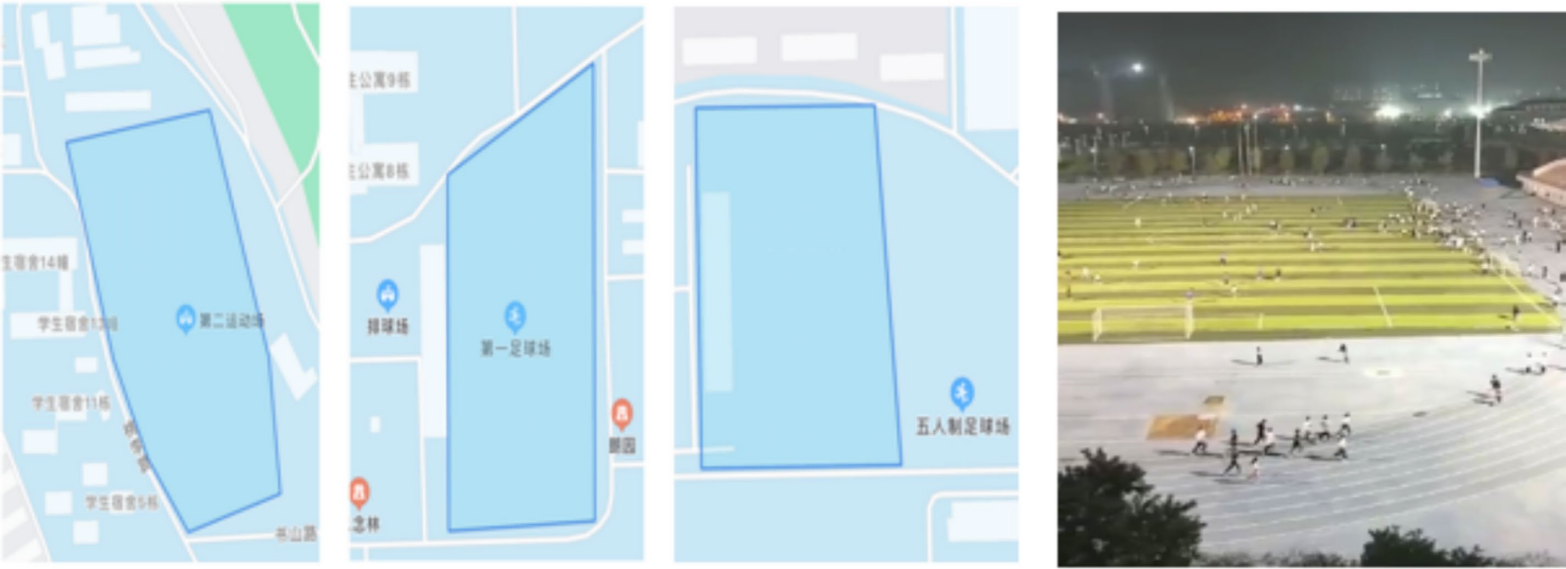
H colleges’ and universities’ sunshine run regulation area and onsite photos.

The “mandatory exercise” policy of H college and university selected in this study is as follows: non-sports major undergraduates in their freshman and sophomore years must run 2 kilometers within the specified time and area (Figure 2) from the 5th week to the 16th week, with a total of no less than 38 times to be qualified. The effective running range for men is as follows: 1 km/3–8 min, the step requirement is at least 700 steps for 1 km and at least 1,400 steps for 2 km; the effective running range for women is as follows: 1 km/3–9 min, the step requirement is at least 700 steps for 1 km and at least 1,400 steps for 2 km; facial recognition will be performed during the run, and it must be successfully recognized within the specified range and time after hearing the prompt; otherwise, it will not be counted; and students who do not complete the 38 running requirements will receive 0 points for the usual score of the university physical education course. Undergraduate students majoring in sports in their freshman and sophomore years must perform morning exercises from the 2nd week to the 16th week, from Monday to Friday (time: 6:30–7:10). In addition, the results of the colleges’ and universities’ physical fitness tests are combined with the colleges’ and universities’ evaluation and selection of outstanding individuals, which requires sports major students to reach the “excellent” level and public sports students to reach the “qualified” level in the physical fitness test. All the 589 randomly selected participants in this study had experienced 2 years of “mandatory exercise” and 3 months had passed.

### Implementation of mandatory exercise in colleges and universities

The study analyzes the implementation of “mandatory exercise” from different dimensions. In terms of the attitudes of the school in carrying out mandatory exercise, this study randomly sampled 30 teachers from various majors; 80% of the interviewed teachers fully supported the school’s behavior in terms of mandatory exercise, believing that 3–4 exercise events per week are essential and beneficial to the physical and mental health of students. Another 22% of the interviewed teachers believe that it is necessary to guide students to exercise, but the management should be further scientific and humanized, such as sports major students who cannot participate in sports due to injuries; even if the hospital has issued a sports obstacle certificate, it is unreasonable for the school to only recognize it as “qualified.” For such students who cannot obtain an “excellent” level, canceling their qualifications for evaluation and selection of outstanding individuals is obviously unreasonable. Among the 589 randomly selected junior students, 65% students fully supported the school’s behavior in carrying out mandatory exercise, believing that they could easily complete the exercise tasks. Twenty-two percent of the interviewed students expressed support for the school’s behavior in carrying out mandatory exercise, but they had some difficulties in completing the tasks. Eleven percent of the interviewed students expressed not fully supporting the school’s behavior in carrying out mandatory exercise, believing that it is challenging to complete the tasks or do not like mandatory exercise or believing that the design of mandatory exercise is unreasonable and that there are multiple repeated tests in the content of sunshine running, physical fitness tests, and physical education classes. Two percent of the interviewed students expressed their dislike for the school’s behavior in carrying out mandatory exercise, believing that extracurricular activities are what students can freely arrange outside of teaching and that the school should not interfere too much. In terms of content, 87% of the students believed that the school should not only engage in mandatory exercise in running but also expand the content of activities and optimize the top-level policy design of mandatory exercise according to students’ interests and hobbies. With respect to students’ sense of gain from mandatory exercise, 63% of the interviewed students believed that the gain was very large, 35% of the interviewed students believed that the gain was relatively large, and only 2% of the interviewed high school students believed that the gain was not large. Overall, most of the interviewed students highly evaluated the school’s mandatory exercise and held a positive attitude. However, compared with the feedback from the interviewed students, there is also a scientific basis, and the school still needs to further improve the level of mandatory exercise content and continuously strengthen the human care of exercise and the effectiveness evaluation of the school’s “mandatory exercise.”

### Impact of mandatory exercise on exercise habits

#### Correlation analysis

The results of the Pearson correlation analysis (Table 1) revealed that the Pearson correlation coefficients between the two variables of mandatory exercise, self-efficacy, exercise attitudes, and exercise habits were or above 0.457, indicating a moderate to high correlation, and were statistically significant. This finding indicates that there is a certain covariation relationship between mandatory exercise, self-efficacy, exercise attitudes, and exercise habits; that is, as the development of mandatory exercise increases, the level of college and university students’ self-efficacy increases, exercise attitudes also improve, and the promotion of exercise habit formation improves. The significant correlation between the above variables further highlights the importance of the development of colleges’ and universities’ mandatory exercise in promoting college and university students’ self-efficacy and exercise attitudes, which is a predisposing factor for students to develop exercise habits.

**Table 1:** Pearson correlation analysis results between mandatory exercise, self-efficacy, exercise attitude, and exercise habits (standardized parameters).

Variable	Compelled exercise	Self-efficacy	Exercise attitude	Exercise habit
Mandatory exercise	1			
Self-efficacy	0.457**	1		
Exercise attitude	0.489**	0.737**	1	
Exercise habit	0.467**	0.498**	0.548**	1

* indicates *p* < 0.05, ** indicates *p* < 0.01, *** indicates *p* < 0.001; all variables are converted to a 5-point scale for calculation.

## Multiple mediating effect analysis

Using Hayes’s SPSS plugin Process V4.0 Model 6, by controlling for variables such as gender and hobbies, the multiple mediating effects of self-efficacy and exercise attitudes on the relationship between college and university mandatory exercise and exercise habits were analyzed and tested (Table 2). Model 1 takes self-efficacy as the dependent variable to examine the independent effect of mandatory exercise while controlling for related variables. The impact of college and university mandatory exercise on self-efficacy is significant (B = 0.6495, *p* < 0.001); That is, for each unit increase in the mandatory exercise effect of schools, students’ self-efficacy also increases by 0.6495 units, showing a positive predictive effect. Model 2 takes exercise attitude as the dependent variable to test the impact of mandatory exercise and self-efficacy on exercise attitude. College and university-based mandatory exercise had a significant positive predictive effect on exercise attitudes (*B* = 1.564, *p* < 0.001); that is, for each unit increase in mandatory exercise, the exercise attitude score increased by 1.564. Self-efficacy also had a significant positive predictive effect on exercise attitudes (*B* = 0.3593, *p* < 0.001); that is, for each unit increase in self-efficacy, the exercise attitude score increased by 0.3593. Model 3 is the main model of this study, which takes exercise habits as the dependent variable to test the impact of college and university mandatory exercise, self-efficacy, and exercise attitudes on exercise habits. With respect to the control variables, there was no significant difference in the impact of sex or hobbies on exercise habits.

**Table 2:** Regression analysis results for the mediating effect of self-efficacy and exercise attitude on the relationship between mandatory exercise and exercise habit (*n* = 589).

Variable	Model 1: Self-efficacy		Model 2: Exercise attitude		Model 3: Exercise habit	
	Coefficient	SE	Coefficient	SE	Coefficient	SE
Mandatory exercise	0.6495***	0.0597	0.1564***	0.028	0.4690***	0.0755
Self-efficacy			0.3593***	0.0177	0.1907**	0.0607
Exercise attitude					0.6271***	0.1085
Gender	−0.0391	0.0662	0.002	0.0284	0.0396	0.0744
Hobby	−0.3006***	0.0684	−0.1598***	0.0298	−0.1074	0.08
Constant	3.0279***	0.1772	2.0219	0.093	0.4616	0.3281
R^2^	0.2429		0.5961		0.3652	
*F* value	62.5465***		215.4329***		67.0782***	

* indicates *p* < 0.05, ** indicates *p* < 0.01, *** indicates *p* < 0.001; all variables are converted to a 5-point scale for calculation.

**Figure 3:**
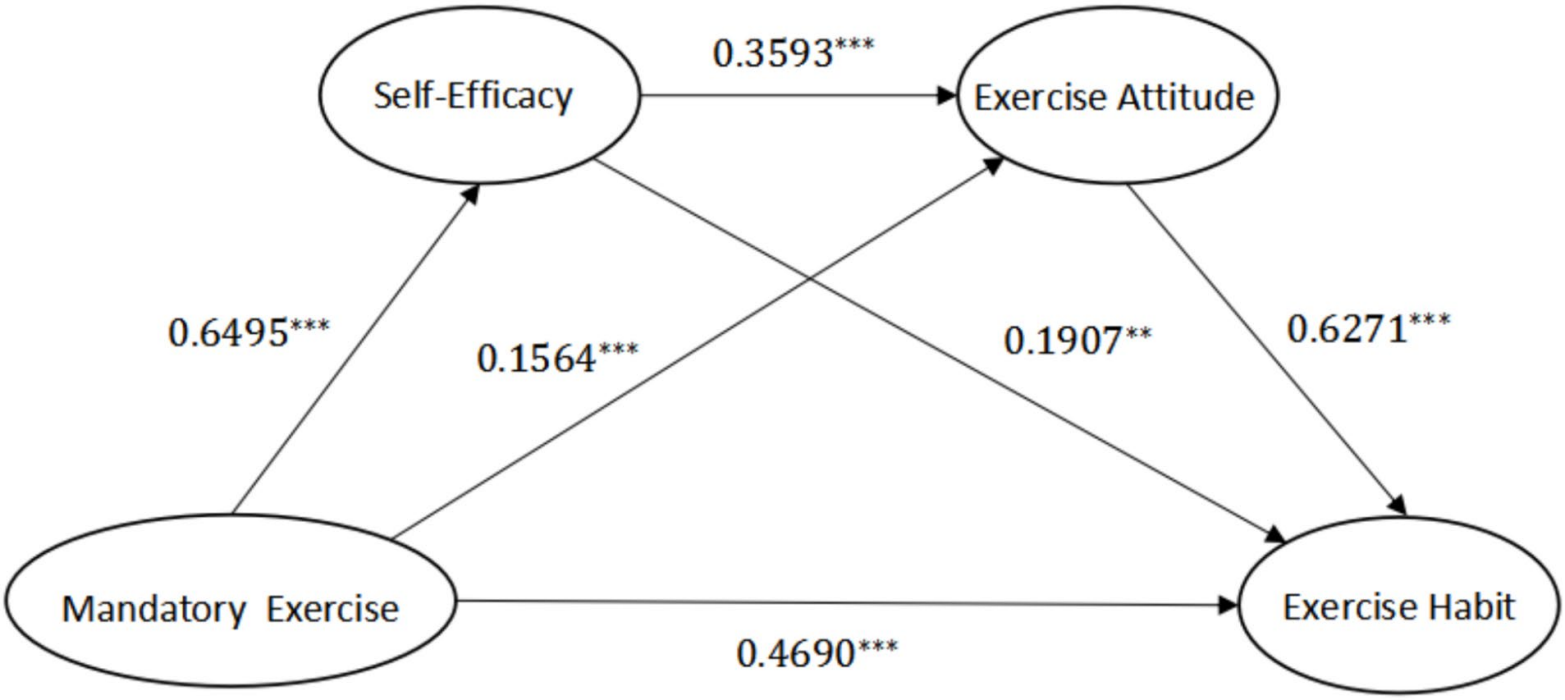
Path coefficients of the multiple mediating regression model between mandatory exercise and exercise habits. * indicates *p* < 0.05, ** indicates *p* < 0.01, *** indicates *p* < 0.001; all variables are converted to a 5-point scale for calculation.

According to Model 3, after adding the two mediating variables of self-efficacy and exercise attitude, the direct predictive effect of mandatory exercise on exercise habits was still significant (*B* = 0.4690, *p* < 0.001); that is, after controlling for the mediating variables and related covariates, for each unit increase in mandatory exercise, the score of the exercise habit variable increased by 0.4690. Research Hypothesis 1 is supported. In addition, the positive effects of self-efficacy (*B* = 0.1907, *p* < 0.01) and exercise attitudes (*B* = 0.6271, *p* < 0.001) on exercise habits are also significant. For each unit increase in college and university students’ self-efficacy, the score of the exercise habit variable increases by 0.1907; for each unit increase in college and university students’ exercise attitudes, the score of the exercise habit variable increases by 0.6271. The comprehensive results show that mandatory exercise in colleges and universities positively predicts students’ self-efficacy. The implementation of mandatory exercise has a promoting effect on college and university students’ self-efficacy. Mandatory exercise conducted by colleges and universities and students’ self-efficacy positively predict students’ exercise attitudes. The implementation of mandatory exercise and students’ sense of self-efficacy can foster their exercise attitudes. The implementation of mandatory exercise activities in colleges and universities, along with students’ self-efficacy and exercise attitudes, positively predicts the comprehensive development of students. The better the implementation of mandatory exercise is in colleges and universities, the better the self-efficacy and exercise attitudes of students, and the higher the scores of students’ exercise habits. The path coefficients of the multiple mediation regression model can are shown in Figure 3.

This study uses the bias correction nonparametric percentile bootstrap method, and after 5,000 repeated samplings, the 95% confidence interval is calculated to test the multiple mediating effects (Zhou et al., 2024). According to Table 3, the direct effects of college and university mandatory exercise on students’ exercise habits, the independent mediating effects of self-efficacy and exercise attitudes, and the chain mediating effects of self-efficacy and exercise attitudes, the upper and lower limits of the bootstrap 95% confidence interval do not include 0, indicating that self-efficacy and exercise attitudes play a significant mediating role between mandatory exercise and students’ exercise habits; that is, college and university mandatory exercise not only directly affects the formation of students’ exercise habits but also affects the formation of students’ exercise habits through the independent mediating effects and chain mediating effects of self-efficacy and exercise attitudes. Research Hypothesis 2 and Research Hypothesis 3 are supported. In addition, according to Table 3, the direct effect of mandatory exercise on college and university students’ exercise habits is 0.4690, the mediating effect of self-efficacy is 0.1239, the mediating effect of exercise attitudes is 0.0981, and the chain mediating effect of self-efficacy and exercise attitudes is 0.1463. The total mediating effect of the above three paths is 0.3683, accounting for 43.99% of the total effect. Compared with the mediating effect of exercise attitudes, the mediating effect of self-efficacy is greater.

**Table 3:** Mediation effect analysis of the relationship between mandatory exercise and exercise habits in colleges and universities.

Influence path	Effect value	Boot S.E.	Boot CI lower	Boot CI upper	Relative effect proportion/%
1. Direct effect (mandatory exercise - exercise habits)	0.469	0.0755	0.3208	0.6173	56.01
2. Mediating effect of self-efficacy	0.1239	0.0448	0.0373	0.216	14.8
3. Mediating effect of exercise attitude	0.0981	0.0261	0.052	0.1551	11.72
4. Serial mediating effect of self-efficacy and exercise attitude	0.1463	0.0318	0.0885	0.2137	17.47
Total mediating effect	0.3683	0.0481	0.2796	0.4666	43.99
Total effect	0.8373	0.0732	0.6936	0.9811	—

## Conclusion

The mandatory exercise of colleges and universities has a significant positive effect on the formation of exercise habits, and self-efficacy and exercise attitudes play significant independent mediating roles and chain mediating effects between mandatory exercise and exercise habits, among which the direct effect is the most significant, followed by the chain mediating effect, the independent mediating effect of self-efficacy, and the independent mediating effect of exercise attitudes. This study further reveals the internal mechanism between mandatory exercise and exercise habits, which is highly important for promoting the formation of exercise habits and the overall development of college and university students.

Future research should focus on verifying the dose–response effects of mandatory exercise (such as the dose–response curve shapes and mediating effects of different intensity, frequency, and duration of mandatory exercise programs on exercise habit development), boundary conditions between direct effects and mediating effects (such as how individual traits and environmental factors regulate the relative weight of direct effects and mediating effects), and inhibitory effects of negative emotions (such as whether temporary negative emotions induced by mandatory exercise weaken the mediating effect of self-efficacy).

## Data Availability

The original contributions presented in the study are included in the article/supplementary material, further inquiries can be directed to the corresponding author.
